# Subcutaneous Sweet’s syndrome associated with the onset of Behcet’s disease^☆^^☆☆^

**DOI:** 10.1016/j.abd.2020.07.021

**Published:** 2021-07-14

**Authors:** Pablo Vargas-Mora, Fernando Valenzuela, Viera Kaplan, Laura Carreño

**Affiliations:** a Department of Dermatology, Faculty of Medicine, Universidad de Chile, Santiago, Chile.; b Pathology Service, Hospital Clínico Universidad de Chile, Santiago, Chile.

Dear Editor,

Sweet Syndrome (SS) is the most frequent of neutrophilic dermatoses.^1^ Some rare variants have been described, such as subcutaneous SS, which clinically usually presents with inflammatory nodules similar to erythema nodosum.^1^, ^2^ Behçet’s Disease (BD) is a multisystemic disease with many symptoms, presenting papules, pustules, or erythema nodosum-like lesions on the skin.^3^

We present a case of subcutaneous SS and BD in the same patient, given its extremely rare association.

A healthy 72-year-old woman consults for painful erythematous nodules in the extremities of two weeks’ evolution, associated with fever and arthralgias. Physical examination reveals erythematous subcutaneous nodules on the extremities, arthritis, and oral ulcers (Figure 1, Figure 2). Laboratory tests: hemogram of 5,850 leucocytes/L (80% neutrophils), erythrocyte sedimentation rate 70 mm/hr, C-reactive protein 158 mL/L. Glycemia, hepatic, renal and thyroid functions are normal. Viral serology (Hepatitis B and C, HIV), blood and urine cultures, *Mycoplasma pneumoniae* serology, autoimmunity studies all negative. A skin biopsy revealed neutrophilic dermohypodermitis, mixed panniculitis, with vasculitis of the medium-small vessels (Fig. 3). With these findings, a diagnosis of subcutaneous SS was postulated, and prednisone 0.5 mg/kg/day was indicated. The patient evolved with no fever and there was complete resolution of the cutaneous lesions after 72 hours of treatment. However, she evolves with a red-eye involvement. Ophthalmic evaluation confirmed intermediate uveitis. HLA-B51 was positive. Given these findings, added to the history of recurrent oral ulcers, BD was diagnosed, and treatment was begun with monthly pulses of cyclophosphamide 300 mg. There was a favorable evolution and complete remission after the first pulse.

**Figure 1:**
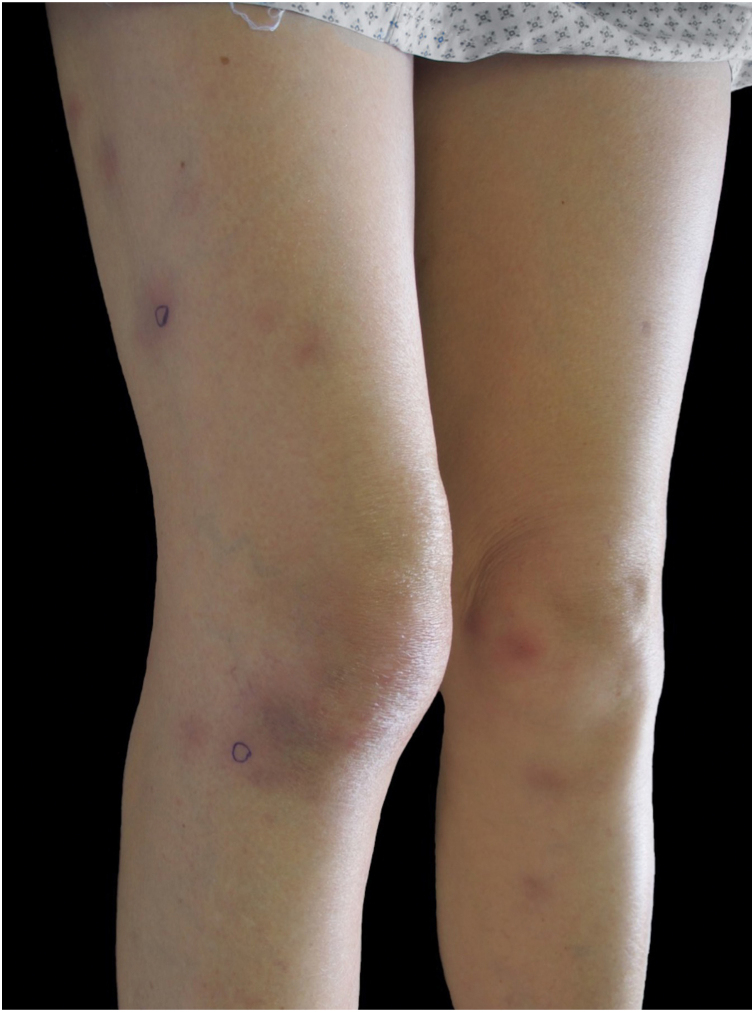
Erythematous subcutaneous nodules in thighs and legs.

**Figure 2:**
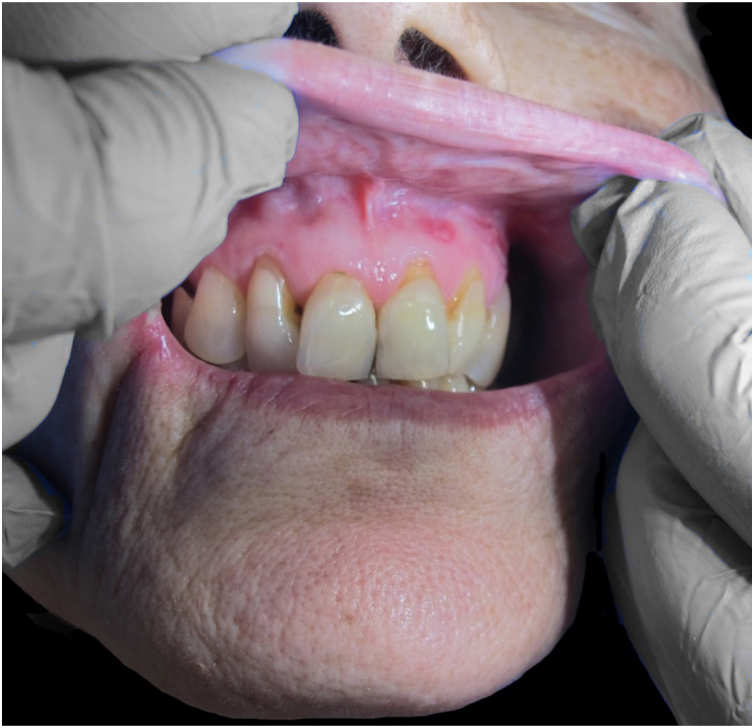
Oral ulcers.

**Figure 3:**
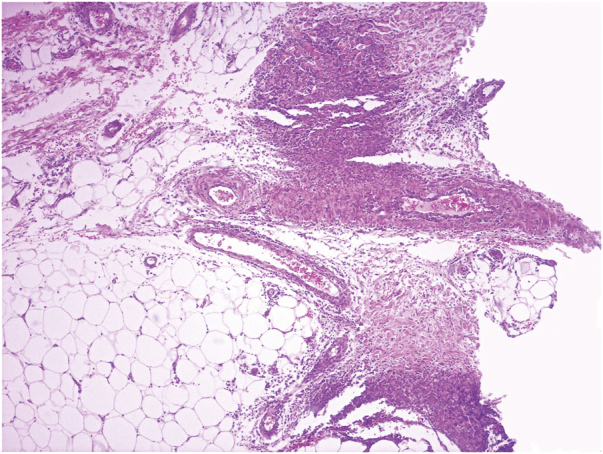
Neutrophilic inflammatory infiltrate in the hypodermis and vascular wall, (Hematoxylin & eosin, ×40).

Subcutaneous SS fulfilled the same spectrum of neutrophilic panniculitis.^1^, ^2^ Our patient complied with the modified diagnostic criteria of SS (Su and Liu)^1^ and also complied with the diagnostic criteria of BD (International Study Group).^3^

There are few reports on the association of both dermatoses.^3^ According to the literature reviewed, the present report is the first case of the subcutaneous variant of SS associated with BD. In histopathological series of SS, the presence of vasculitis has been reported in up to 73% of cases.^4^ On the other hand, series of 26 cases of erythema nodosum-like in the context of BD, it was found that 73% had no classical findings of erythema nodosum, but rather mixed or lobular panniculitis with the presence of vasculitis and in several cases an abundant infiltrate of neutrophils.^5^ This suggests that some of these patients might comply with the criteria for subcutaneous SS, and that the superimposition of BD could lead to SS being under-diagnosed.

We think that the relationship between both diseases is more than a mere coincidence. SS might represent a marker for Behçet’s activity, as in the majority of reported cases it presents in the acute phase or prior to an exacerbation, which is correlated to the clinical evolution of our patient.^5^

Future studies are needed aiming at the wide clinical variability of both diseases and the way they relate to each other, which is probably in a continuum within the spectrum of the still poorly understood neutrophilic dermatoses.

## Financial support

None declared.

## Authors’ contributions

Pablo Vargas-Mora: Approval of the final version of the manuscript; conception and planning of the study; elaboration and writing of the manuscript; obtaining, analyzing, and interpreting the data; effective participation in research orientation; critical review of the literature; critical review of the manuscript.

Fernando Valenzuela: Approval of the final version of the manuscript; conception and planning of the study; elaboration and writing of the manuscript; obtaining, analyzing, and interpreting the data; effective participation in research orientation; critical review of the literature; critical review of the manuscript.

Viera Kaplan: Approval of the final version of the manuscript; conception and planning of the study; elaboration and writing of the manuscript; obtaining, analyzing, and interpreting the data; effective participation in research orientation; critical review of the manuscript.

Laura Carreño: Approval of the final version of the manuscript; conception and planning of the study; obtaining, analyzing, and interpreting the data; effective participation in research orientation; critical review of the manuscript.

## Conflicts of interest

None declared.
